# Supplementary material for: Cellular and gene signatures of tumor-infiltrating dendritic cells and natural-killer cells predict prognosis of neuroblastoma
Source: Nat Commun. 2020 Nov 25;11:5992. doi: 10.1038/s41467-020-19781-y (PMC7689423; doi:10.1038/s41467-020-19781-y)
Supplement: Supplementary file 1 — Supplementary Information [file 41467_2020_19781_MOESM1_ESM.pdf]

a

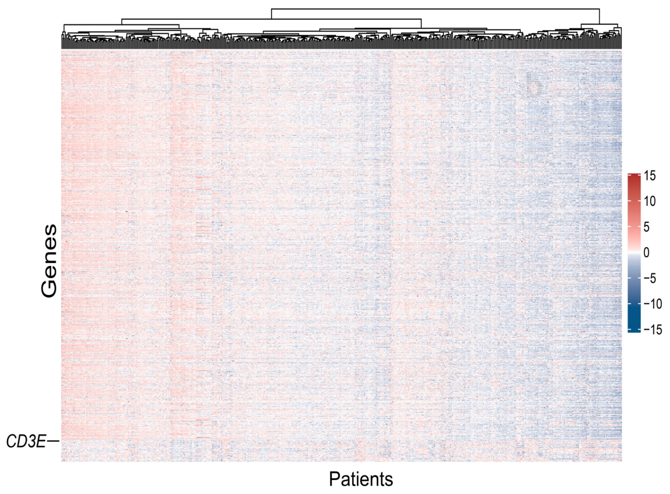

b

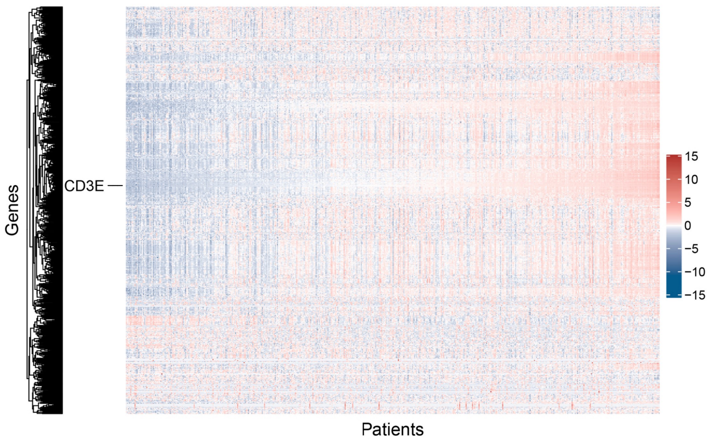

c

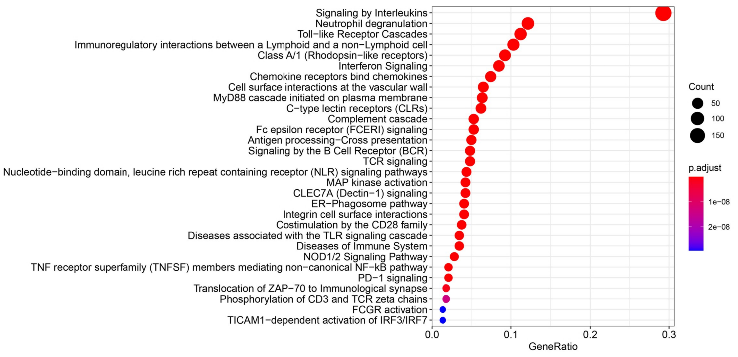

Supplementary Figure 1 related to Figure 1. *CD3E* expression is associated with anti-tumor immune response in human neuroblastoma. a, Heat map and dendrogram of the hierarchical clustering on patients showing the normalized expression of the immune genes in SEQC-NB cohort (n=498). The clustering was done using Euclidean distance with complete linkage method. All genes are on the left side of the heat map and the samples on the top. **b**, Heat map and dendrogram of the hierarchical clustering on genes showing the expression pattern of the immune genes in SEQC-NB cohort (n=498). **c**, Dotplot enrichment map (hypergeometric test, one-sided) showing the Reactome pathways of the main general 30 functional categories. Dot size represents the number of genes in each pathway; Benjamini-Hochberg adjusted p-value: Red < purple < blue.

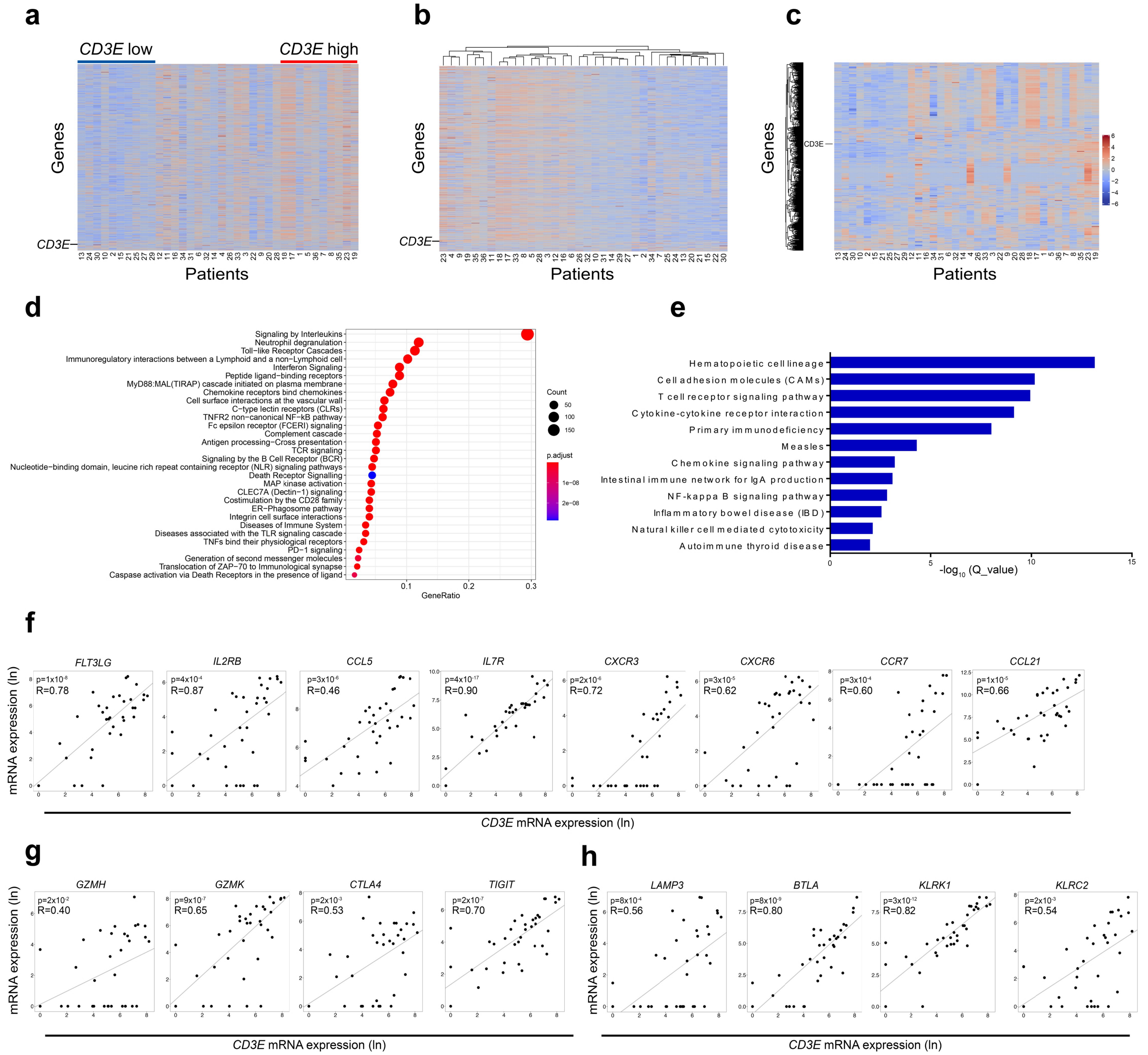

Supplementary Figure 2 related to Figure 1. *CD3E* expression is associated with anti-tumor immune response in human neuroblastoma. a, Heat map of the normalized expression of the immune genes ordered from left to right by increasing levels of *CD3E* expression in Nanostring-NB cohort (n=36). **b**, Heat map and dendrogram of the hierarchical clustering showing the normalized expression of the immune genes in Nanostring-NB cohort (n=36). The clustering was done using Euclidean distance with complete linkage method. All genes are on the left side of the heatmap and the samples on the top. **c**, Heat map and dendrogram of the hierarchical clustering on genes showing the expression pattern of the immune genes in Nanostring-NB cohort (n=36). **d**, Dotplot enrichment map (hypergeometric test, one-sided) showing the Reactome pathways of the main general 30 functional categories. Dot size represents the number of genes in each pathway; Benjamini-Hochberg adjusted p-value: Red < purple < blue. **e**, Gene ontology term enrichment analysis performed by DAVID Bioinformatics Resources (https://david.ncifcrf.gov/) reveals 12 statistically significant (Benjamini-Hochberg adjusted P-value < 0.05) biological process controlled by differentially expressed genes among patients with high *CD3E* expression. **f-h**, Correlation of *CD3E* expression with the indicated genes in Nanostring-NB cohort (n=36). Gene transcripts reported to be associated with increased immune cell infiltration, immune cell trafficking and immune functional status [15, 80, 81, 82] were studied in the SEQC-NB dataset. Genes that met the following criteria: i) a correlation coefficient (R) greater than or equal to at least 0.4 with statistical significance ($p < 0.05$), ii) a significantly different expression between patients with high and low CD3E expression, and iii) showing consensus in the SEQC-NB cohort, were displayed. Robust F-test (two-sided) on the robust regression fit of a linear model was used for data analysis. **f**, Cytokines and chemokines involved in immune cell trafficking. **g**, Activation and exhaustion molecules. **h**, DC and NK function markers. Correlations were assessed measuring the coefficient of determination of a robust linear regression model fit on the data (see Statistical analysis section). No p-value adjustment was required unless otherwise stated. Statistically significant p-values are indicated.

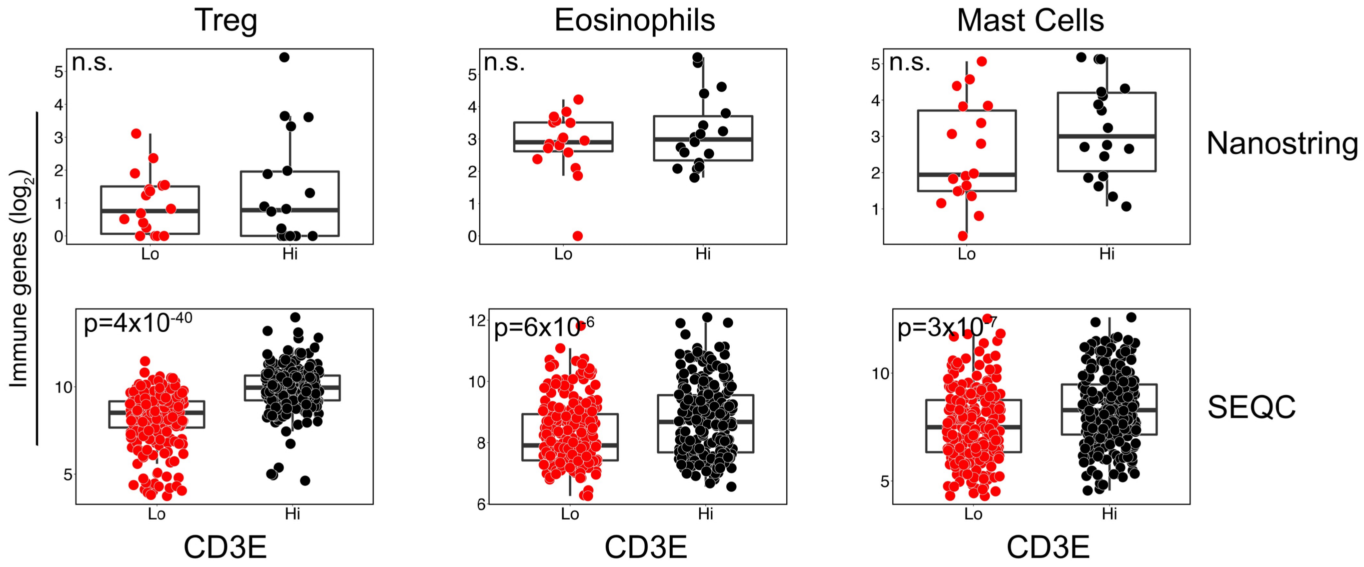

Supplementary Figure 3 related to Figure 2. Intratumoral DCs and NK cells correlate with *CD3E* expression in human neuroblastoma. Box plots of metagene expression values for the indicated immune cell types according to the high (Hi) and low (Lo) levels of *CD3E* mRNA (median split) in primary NB lesions from Nanostring-NB (n=36) and SEQC-NB (n=498) cohorts. The immune cell type scores were calculated as the average expression values of the Nanostring constituent genes. The boxes show the 25th to 75th percentile; the horizontal lines inside the box represent the median; the upper whisker extends to the largest data point, no more than 1.5 times the interquartile range (IQR) from the box; the lower whisker extends to the smallest data point at most 1.5 times the IQR from the box; the dots are individual samples. Data were analyzed by Kruskal-Wallis rank sum test (two-sided). No p-value adjustment was required. Statistically significant p-values are indicated. n.s. = not significant.

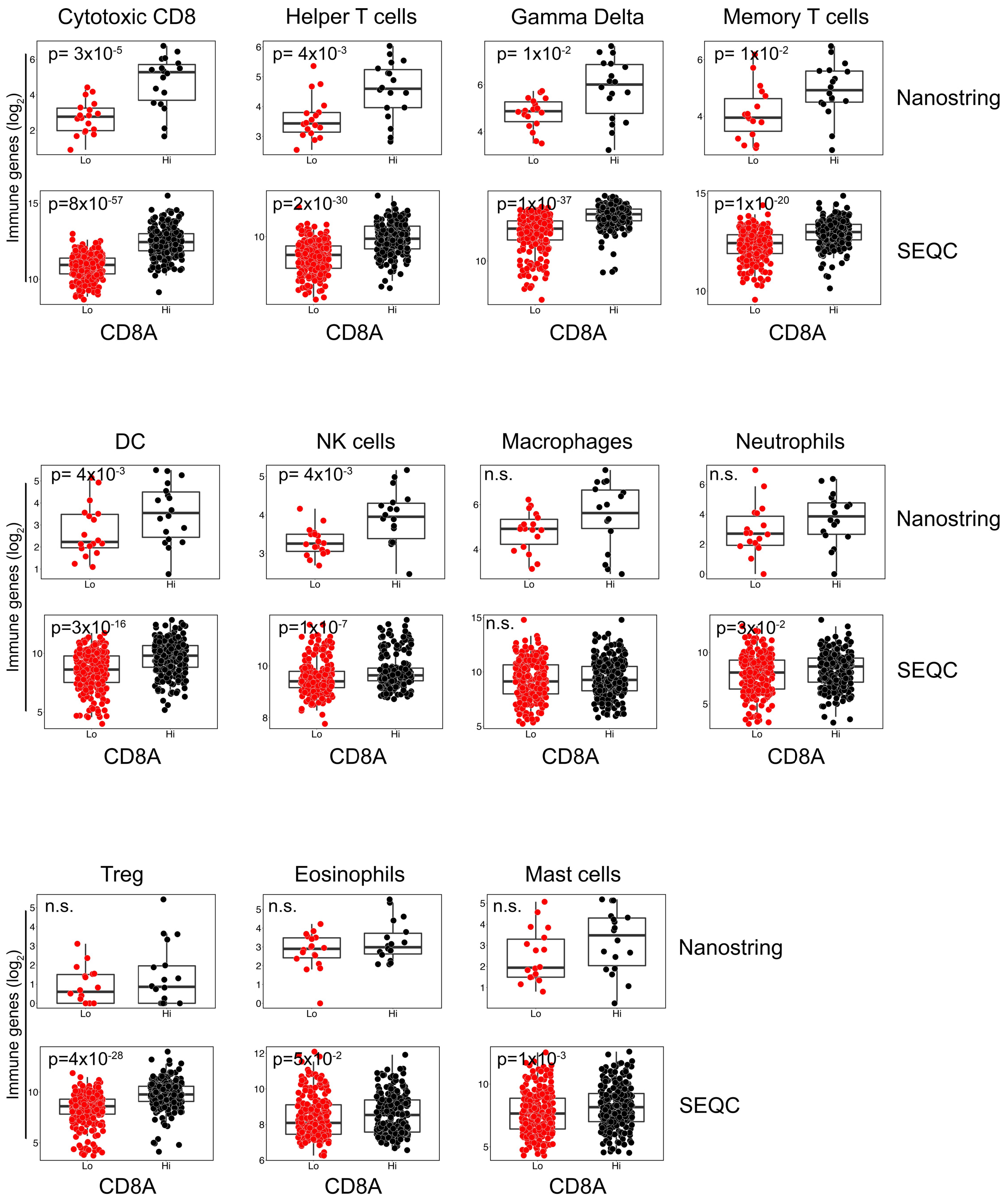

Supplementary Figure 4 related to Figure 2. Intratumoral DCs and NK cells correlate with *CD8A* expression in human neuroblastoma. Box plots of metagene expression values for the indicated immune cell types according to the high (Hi) and low (Lo) levels of *CD8A* mRNA (median split) in primary NB lesions from Nanostring-NB (n=36) and SEQC-NB (n=498) cohorts. The immune cell type scores were calculated as the average expression values of the Nanostring constituent genes. The boxes show the 25th to 75th percentile; the horizontal lines inside the box represent the median; the upper whisker extends to the largest data point, no more than 1.5 times the IQR from the box; the lower whisker extends to the smallest data point at most 1.5 times the IQR from the box; the dots are individual samples. Data were analyzed by Kruskal-Wallis rank sum test (two-sided). No p-value adjustment was required. Statistically significant p-values are indicated. n.s. = not significant.

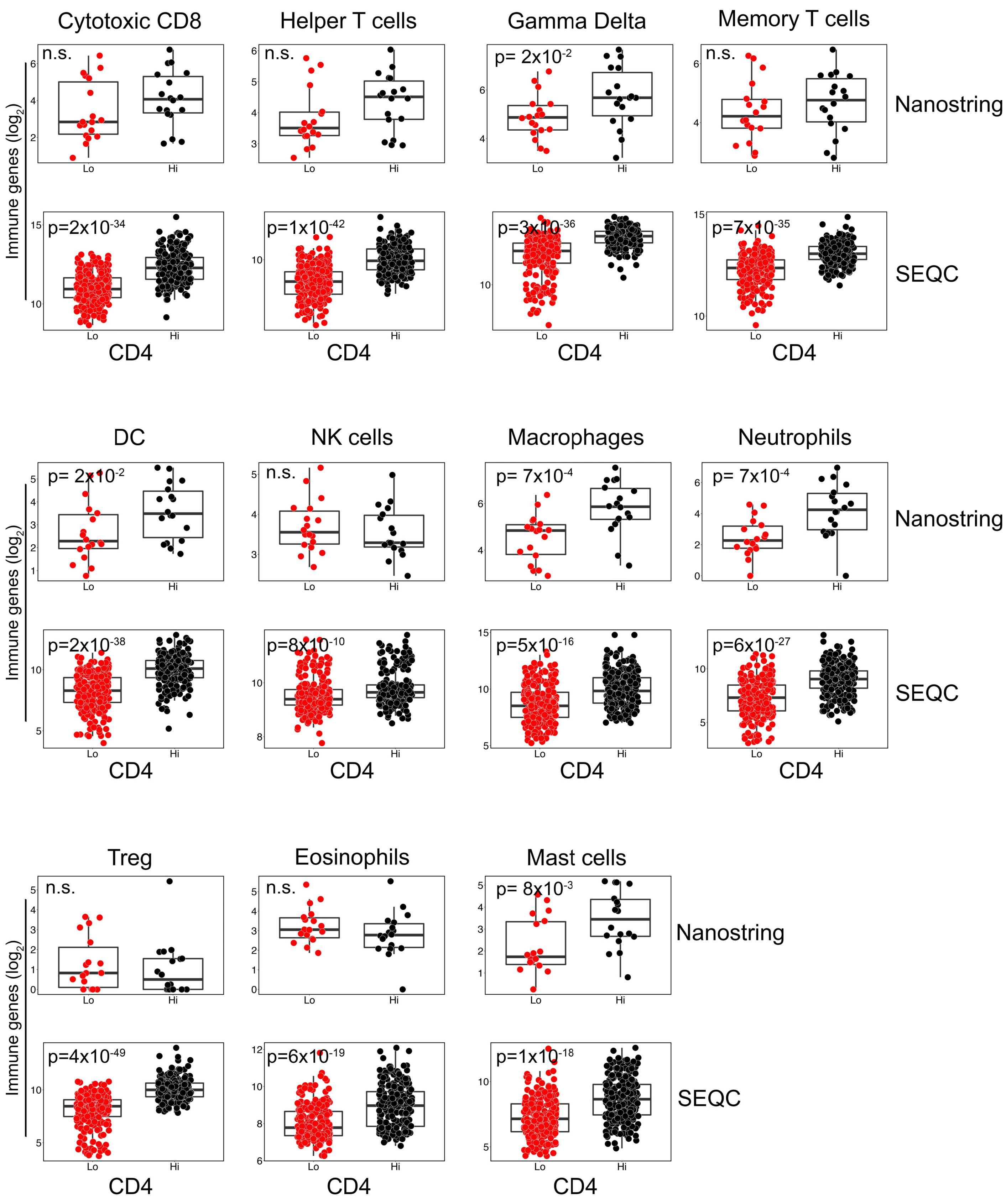

Supplementary Figure 5 related to Figure 2. Intratumoral DCs correlate with *CD4* expression in human neuroblastoma. Box plots of metagene expression values for the indicated immune cell types according to the high (Hi) and low (Lo) levels of *CD4* mRNA (median split) in primary NB lesions from Nanostring-NB (n=36) and SEQC-NB (n=498) cohorts. The immune cell type scores were calculated as the average expression values of the Nanostring constituent genes. The boxes show the 25th to 75th percentile; the horizontal lines inside the box represent the median; the upper whisker extends to the largest data point, no more than 1.5 times the IQR from the box; the lower whisker extends to the smallest data point at most 1.5 times the IQR from the box; the dots are individual samples. Data were analyzed by Kruskal-Wallis rank sum test (two-sided). No p-value adjustment was required. Statistically significant p-values are indicated. n.s. = not significant.

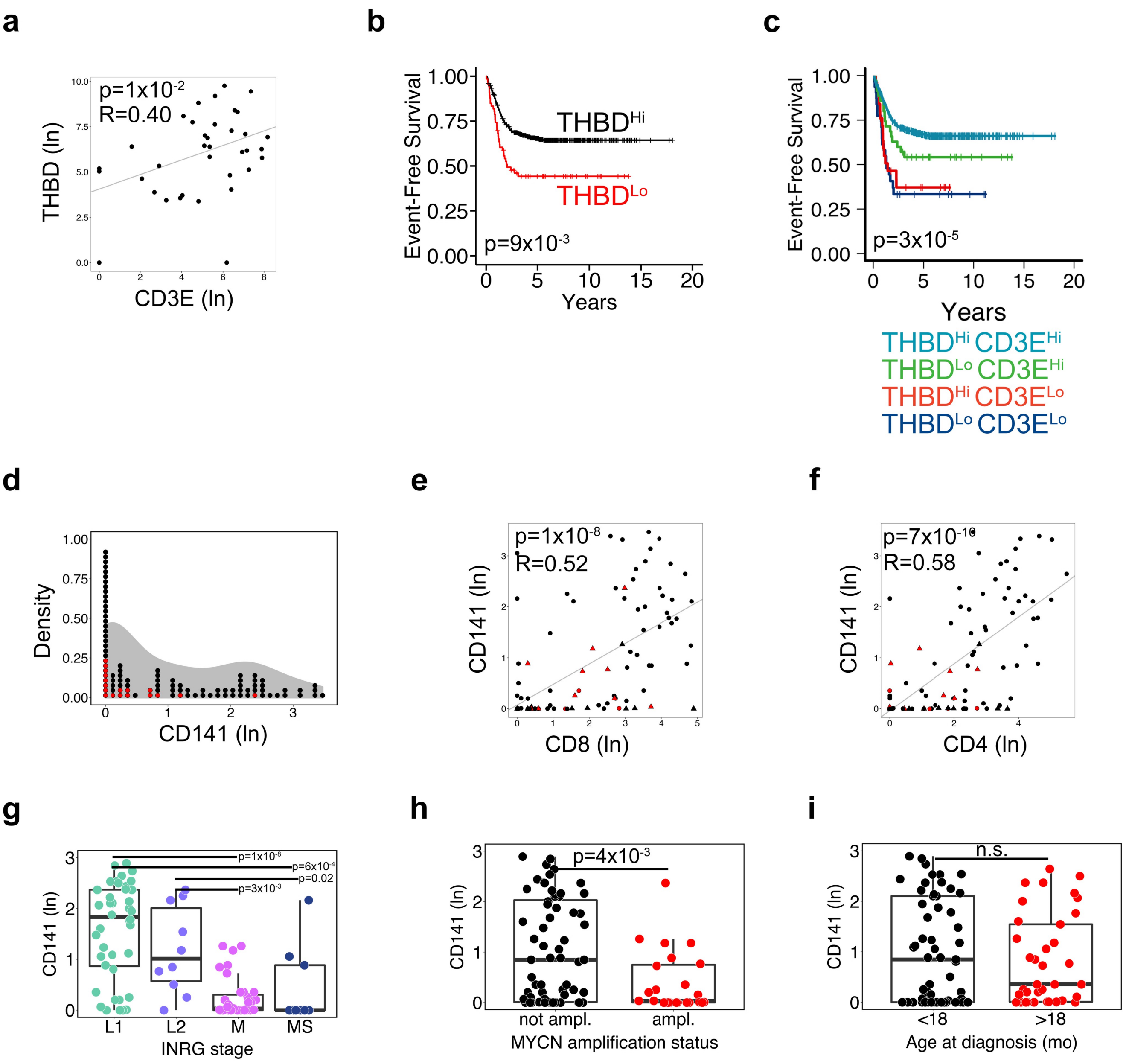

Supplementary Figure 6 related to Figure 3. Intratumoral DC levels correlate with increased overall survival in human neuroblastoma. a, Correlation between *THBD* and *CD3E* gene expression in primary NB lesions from Nanostring-NB (n=36) cohort. Robust F-test (two-sided) was used for data analysis. **b and c,** Kaplan-Meier curves show the duration of event-free survival of NB patients according to the *THBD* gene expression alone (**b**), or in combination with *CD3E* gene (**c**) in SEQC-NB (n=498) cohort. Hi = high. Lo = low. Log-rank test with Miller and Siegmund p-value correction was used. **d**, Density plot of $CD141^{+}$ cells in NB patients. Black and red dots, patients who are alive and dead, respectively. Gray plot, density of $CD141^{+}$ cells in all patients. **e** and **f**, Scatter plots showing the correlation between $CD141^{+}$ and $CD8^{+}$ (**e**) or $CD4^{+}$ (**f**) cell densities in NB patients. Black and red dots, patients who are alive and dead respectively; triangle and dots, *MYCN* amplified and non-amplified patients, respectively. Robust F-test (two-sided) was used for data analysis. **g,** Box plots of the $CD141^{+}$ cell density according to the INRG stages. Dunn's multiple comparisons post-hoc test (two-sided) with Benjamini-Hochberg p-value adjustment was employed. **h** and **i**, Box plots of the $CD141^{+}$ cell density according to the *MYCN* amplification status (not ampl. and ampl. = *MYCN* not amplified and amplified respectively) (**h**) and age at diagnosis (**i**). Data were analyzed by Kruskal-Wallis rank sum test (two-sided). **g-i**, The boxes show the 25th to 75th percentile; the horizontal lines inside the box represent the median; the upper whisker extends to the largest data point, no more than 1.5 times the IQR from the box; the lower whisker extends to the smallest data point at most 1.5 times the IQR from the box; the dots are individual samples. Correlations were assessed measuring the coefficient of determination of a robust linear regression model fit on the data (see Statistical analysis section). **d-i**, data analysis were performed on n=104 biologically independent NB specimens. No adjustment was required unless otherwise stated. Statistically significant p-values are indicated. n.s. = not significant.

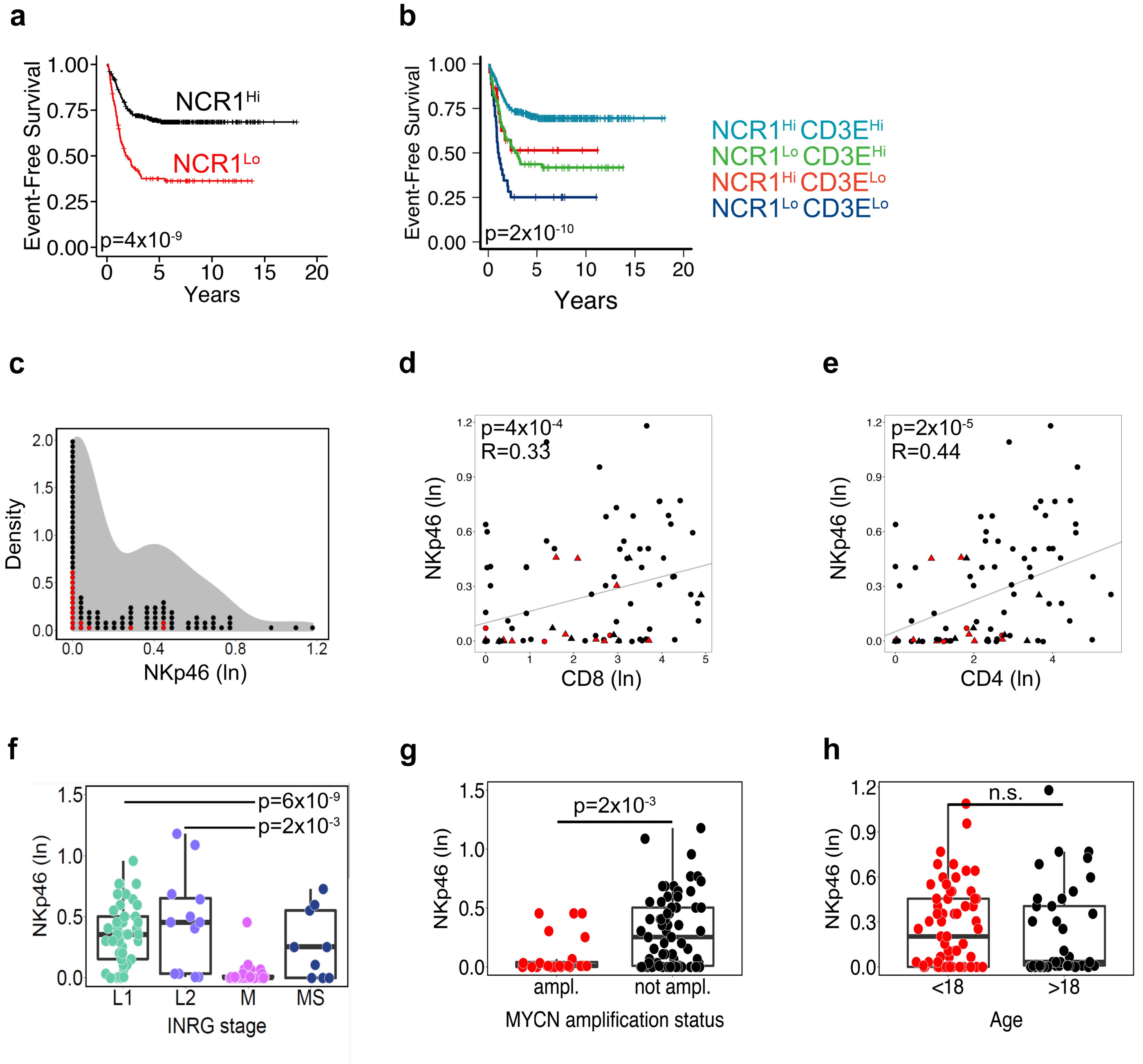

Supplementary Figure 7 related to Figure 4. Intratumoral NK cell levels correlate with increased overall survival in human neuroblastoma. a and b, Kaplan-Meier curves show the duration of event-free survival of NB patients according to the *NCR1* gene expression alone (**a**), or in combination with the *CD3E* gene (**b**) in SEQC-NB (n=498) cohort. Log-rank test with Miller and Siegmund p-value correction was used. Hi = high. Lo = low. **c**, Density plot of NKp46$^+$ cell density in NB patients. Black and red dots indicate alive and dead patients, respectively. Gray plot, density of NKp46$^+$ cells in all patients. **d** and **e**, Scatter plots showing the correlation between NKp46$^+$ and CD8$^+$ (**d**) or CD4$^+$ (**e**) cell densities in NB patients, analyzed by robust F-test (two-sided). Black and red dots, patients who are alive and dead respectively; triangle and dots, *MYCN* amplified and non-amplified patients, respectively. **f**, Box plots of the NKp46$^+$ cell density according to the INRG stages. **g** and **h**, Box plots of the NKp46$^+$ cell density according to the *MYCN* amplification status (not ampl. and ampl. = *MYCN* not amplified and amplified respectively) (**g**) and age at diagnosis (**h**). **f-h**, The boxes show the 25th to 75th percentile; the horizontal lines inside the box represent the median; the upper whisker extends to the largest data point, no more than 1.5 times the IQR from the box; the lower whisker extends to the smallest data point at most 1.5 times the IQR from the box; the dots are individual samples. Correlations were assessed measuring the coefficient of determination of a robust linear regression model fit on the data (see Statistical analysis section). **d-h**, data analysis were performed on n=104 biologically independent NB specimens. No adjustment was required unless otherwise stated. Statistically significant p-values are indicated. n.s. = not significant.

a

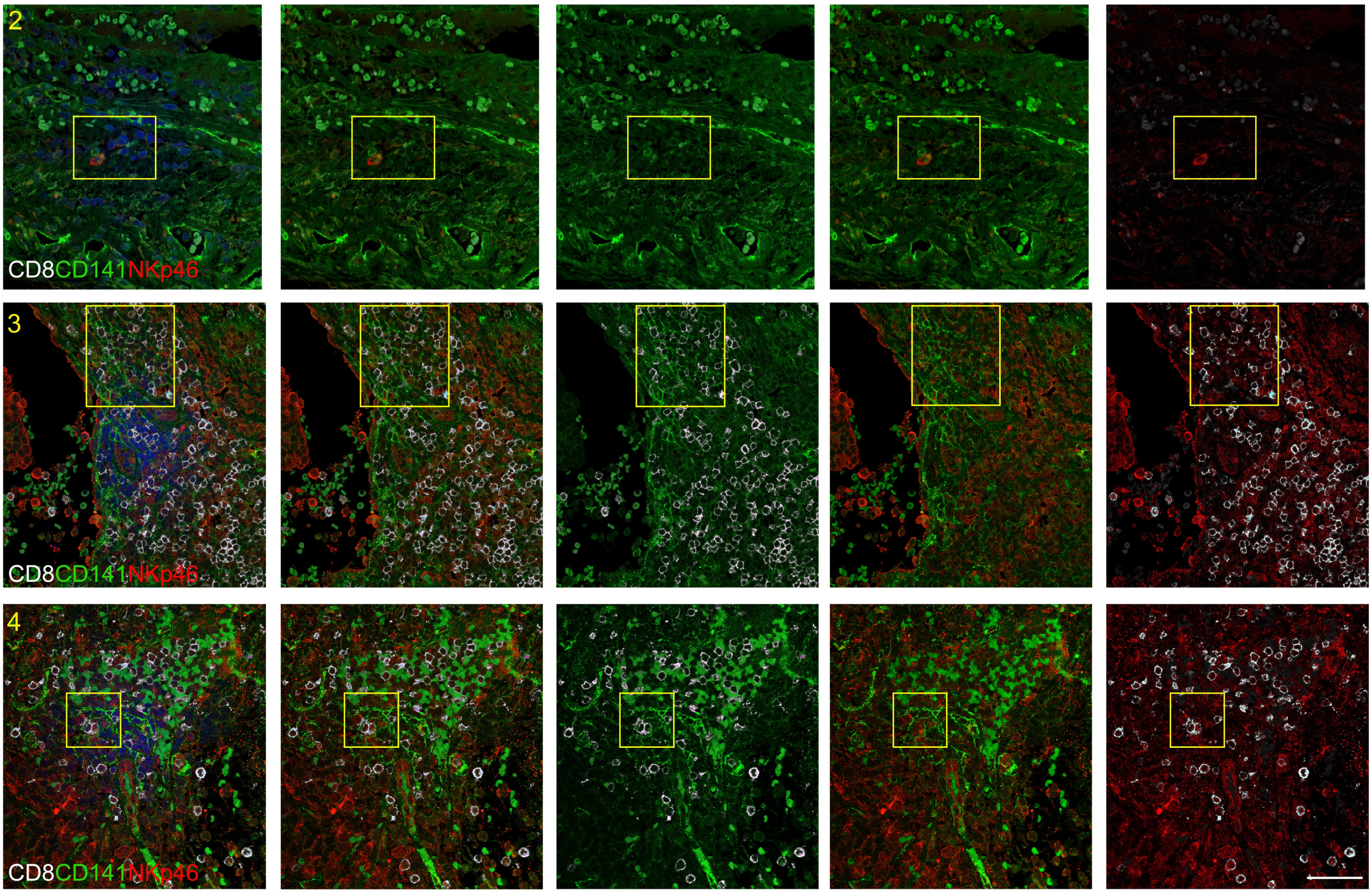

b

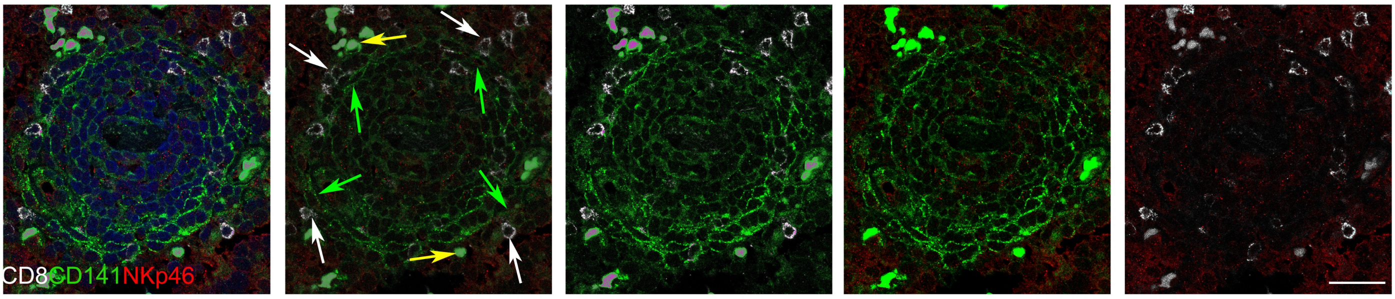

Supplementary Figure 8 related to Figure 5. Cross correlation between intratumoral DCs and NK cells in human neuroblastoma. a, Multiple immunofluorescence staining of NB tumor lesions for CD8 (white), CD141 (green) and NKp46 (red). Squares with solid yellow lines indicate the enlargements reported in Figure 5a for the scenarios 2, 3 and 4 respectively. Scenario 2: tumor nest including CD141$^+$ cells interacting with NKp46$^+$ cells; Scenario 3: lymphocyte aggregate in proximity to a tumor septa in which CD141$^+$ cells interact with CD8$^+$ T cells; Scenario 4: highly infiltrated tumor nest containing CD141$^+$ cells interacting with both CD8$^+$ T and NKp46$^+$ cells. Hoechst stains nuclei blue. Red blood cells are indicated by yellow arrows. **b**, Representative images of immunofluorescence staining for CD8 (white), CD141 (green) and NKp46 (red) in a typical TLS within a primary NB lesion. CD141$^+$ cells are distributed around the TLS and interact with CD8$^+$ cells. No positive staining for NKp46$^+$ cells was detected. Magnification x60, scale bar 30 μm. The representative images were selected from n=7 biologically independent T cell-enriched NB specimens.

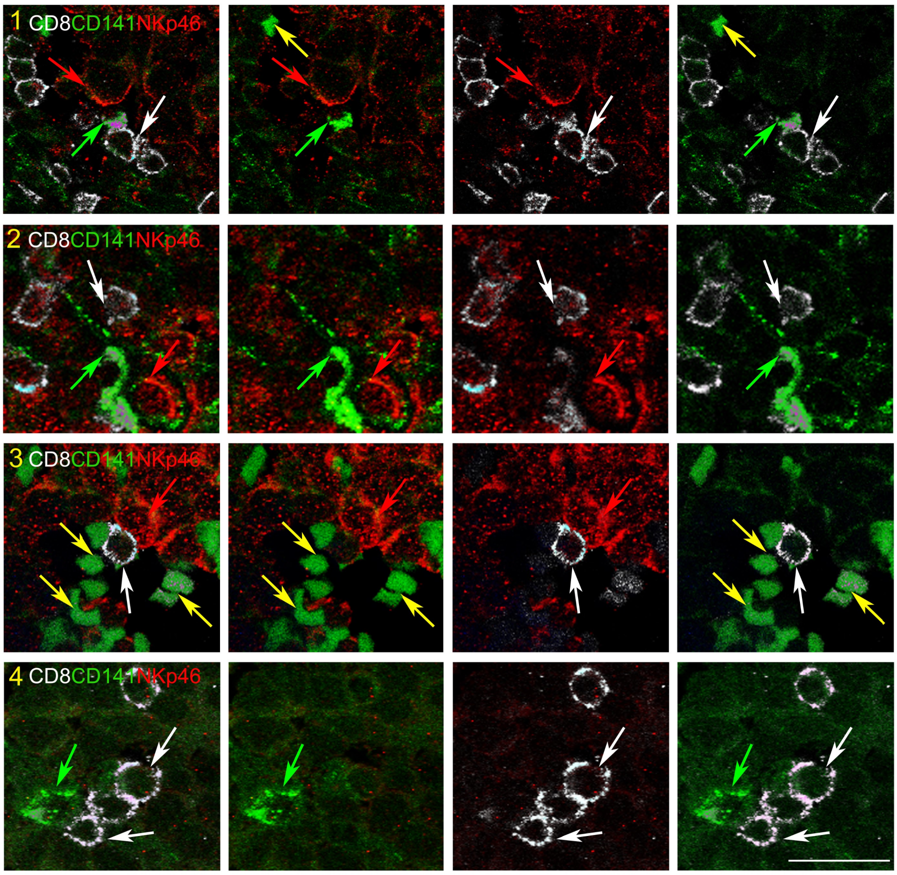

Supplementary Figure 9 related to Figure 5. Cross correlation between intratumoral DCs and NK cells in human neuroblastoma. Additional representative images of multiple immunofluorescence staining within tumor nests of NB lesions for CD8 (white), CD141 (green) and NKp46 (red). Scenario 1 and 2: interactions between $CD8^+$, $CD141^+$ and $NKp46^+$ cells. Scenario 3: interaction between $CD8^+$ and $NKp46^+$ cells. Scenario 4: interaction between $CD8^+$ and $CD141^+$ cells. Red blood cells are indicated by yellow arrows. Magnification x60 (zoom), scale bar 30 µm. The representative images were selected from n=7 biologically independent T cell-enriched NB specimens.

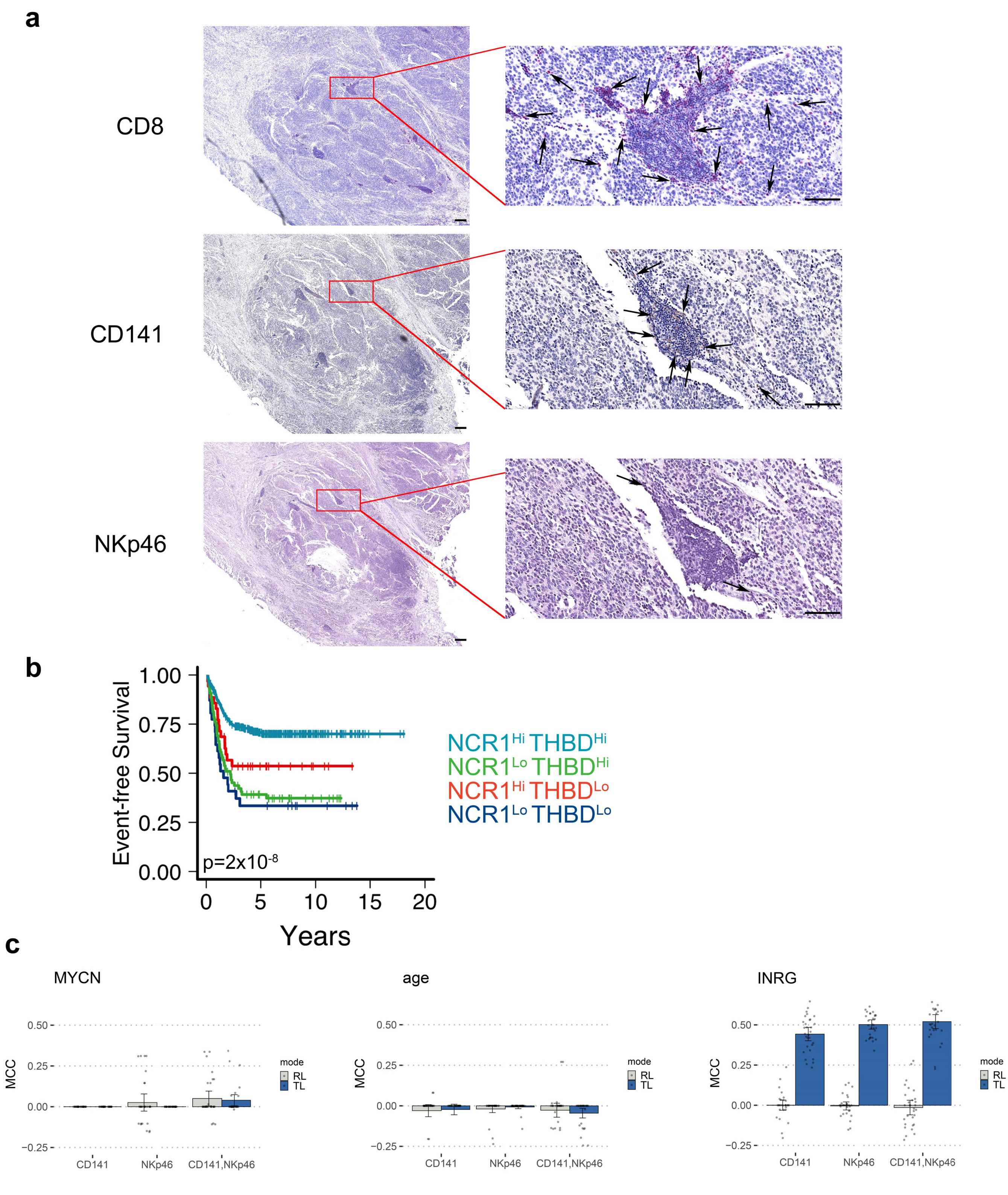

Supplementary Figure 10 related to Figure 5. Cross correlation between intratumoral DCs and NK cells in human neuroblastoma. a, Representative examples of CD8, CD141 and NKp46 immuno-histochemical staining in a highly infiltrated primary NB lesion. On the left, serial tissue sections of the indicated stainings show the typical structures of a NB lesion, including tumor nests, the surrounding tumor septa regions, and the TLSs sparsely scattered around tumor cells. Magnification with x2 on the left and x20 on the right, scale bar 30 µm. Black arrows indicate $CD8^+$ (red), $CD141^+$ (brown) and $NKp46^+$ (red) cells. Nuclei were counterstained with hematoxylin (blue). CD8 and CD141-expressing cells are distributed both within and outside TLSs. NKp46-expressing cells are mainly present within tumor nests in close proximity to or outside the TLSs. The representative images were selected from n=104 biologically independent NB specimens. **b**, Kaplan-Meier curves of event-free survival of NB patients according to the combined expression of *THBD* and *NCR1* genes. Log-rank test with Miller and Siegmund p-value correction was used. Hi = high. Lo = low. **c**, Barplots of average cross-validation MCC values for logistic regression models predicting *MYCN* amplification, age at diagnosis and INRG stage, using as predictors CD141, NKp46, and their combination (n=30 independent data points for each barplot). RL: random labels mode; TL: true labels mode. Black bars represent 95% confidence intervals. Statistically significant p-values are indicated.

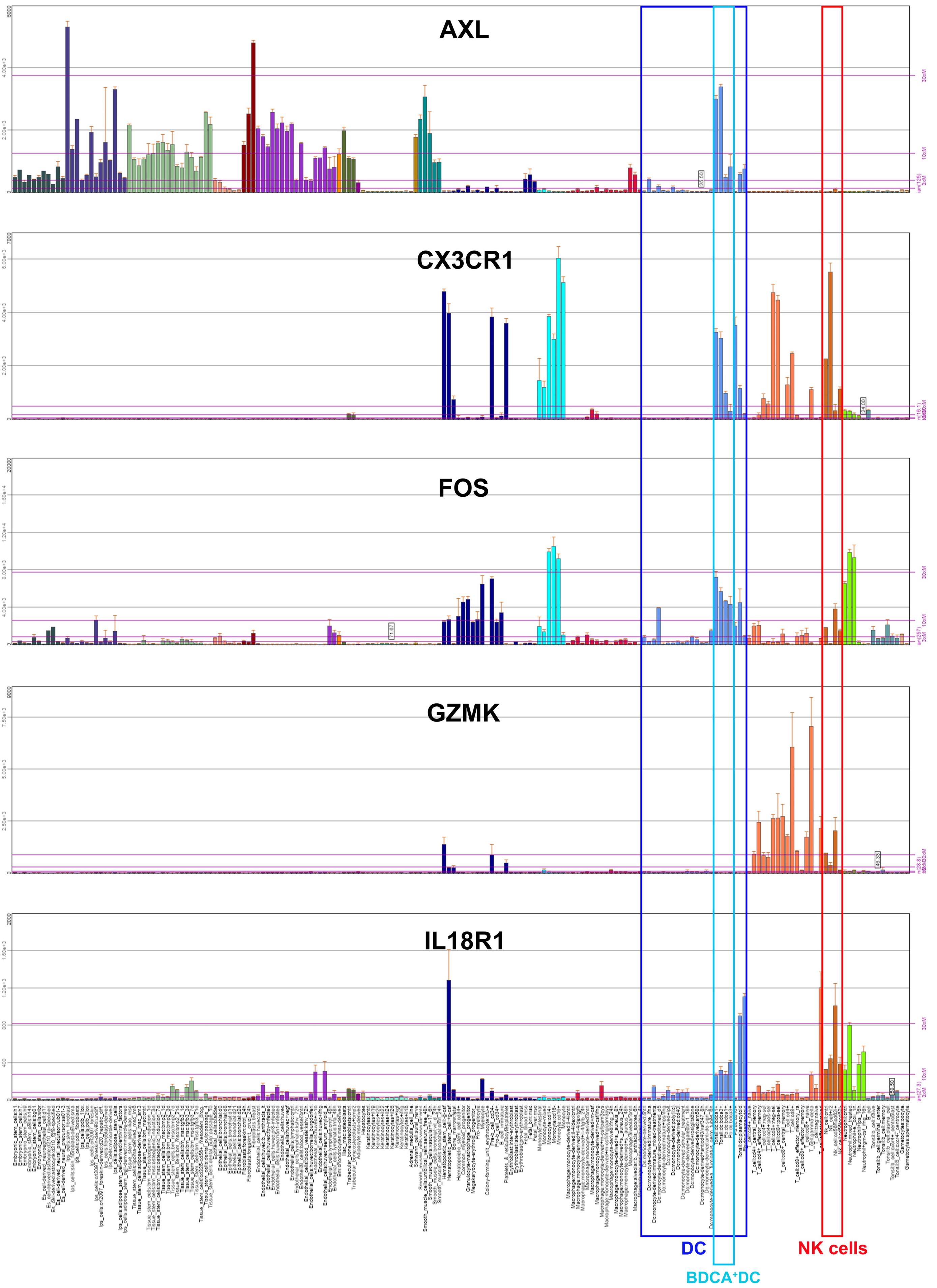

Figure S11 continued

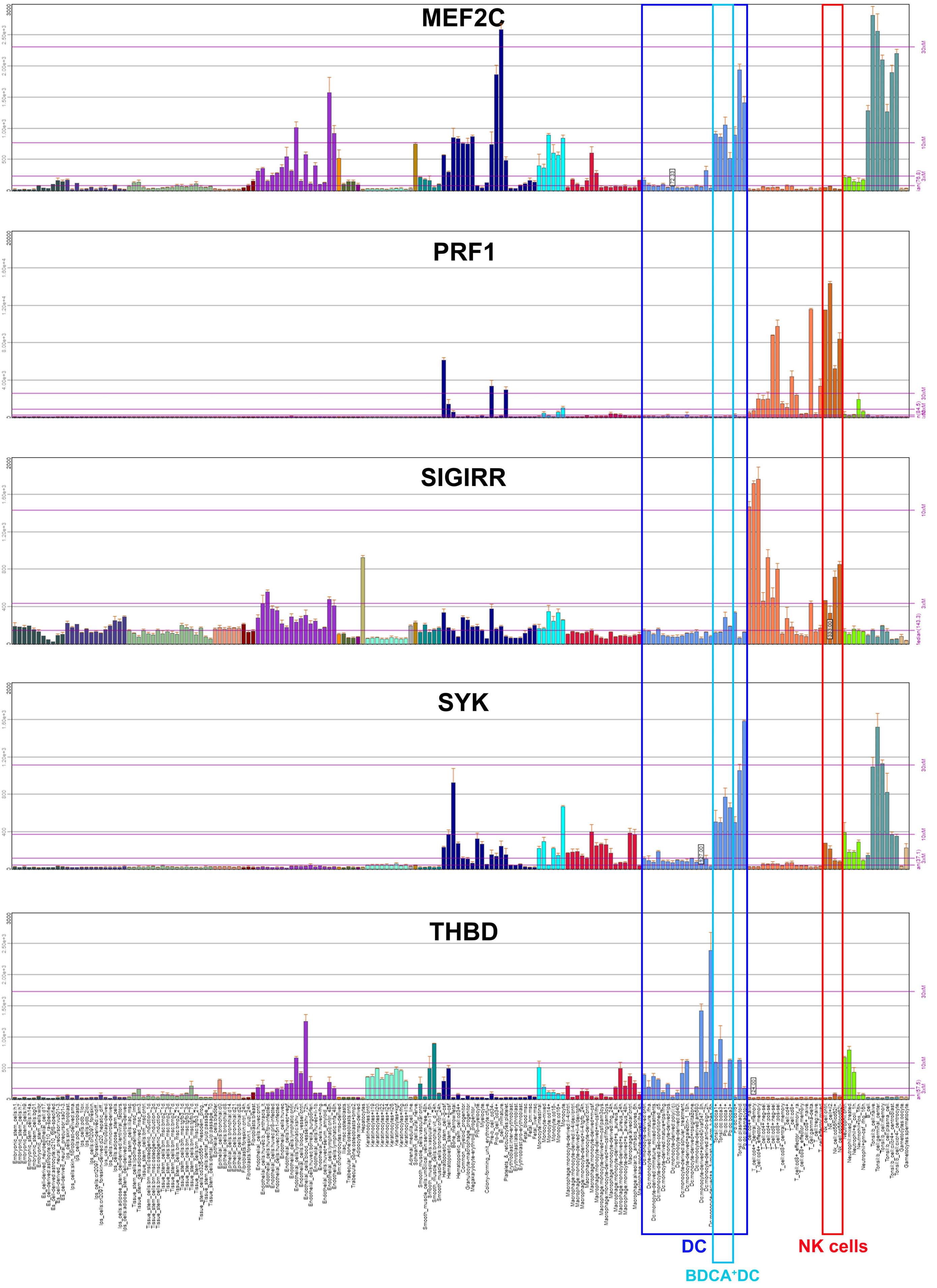

Figure S11 continued

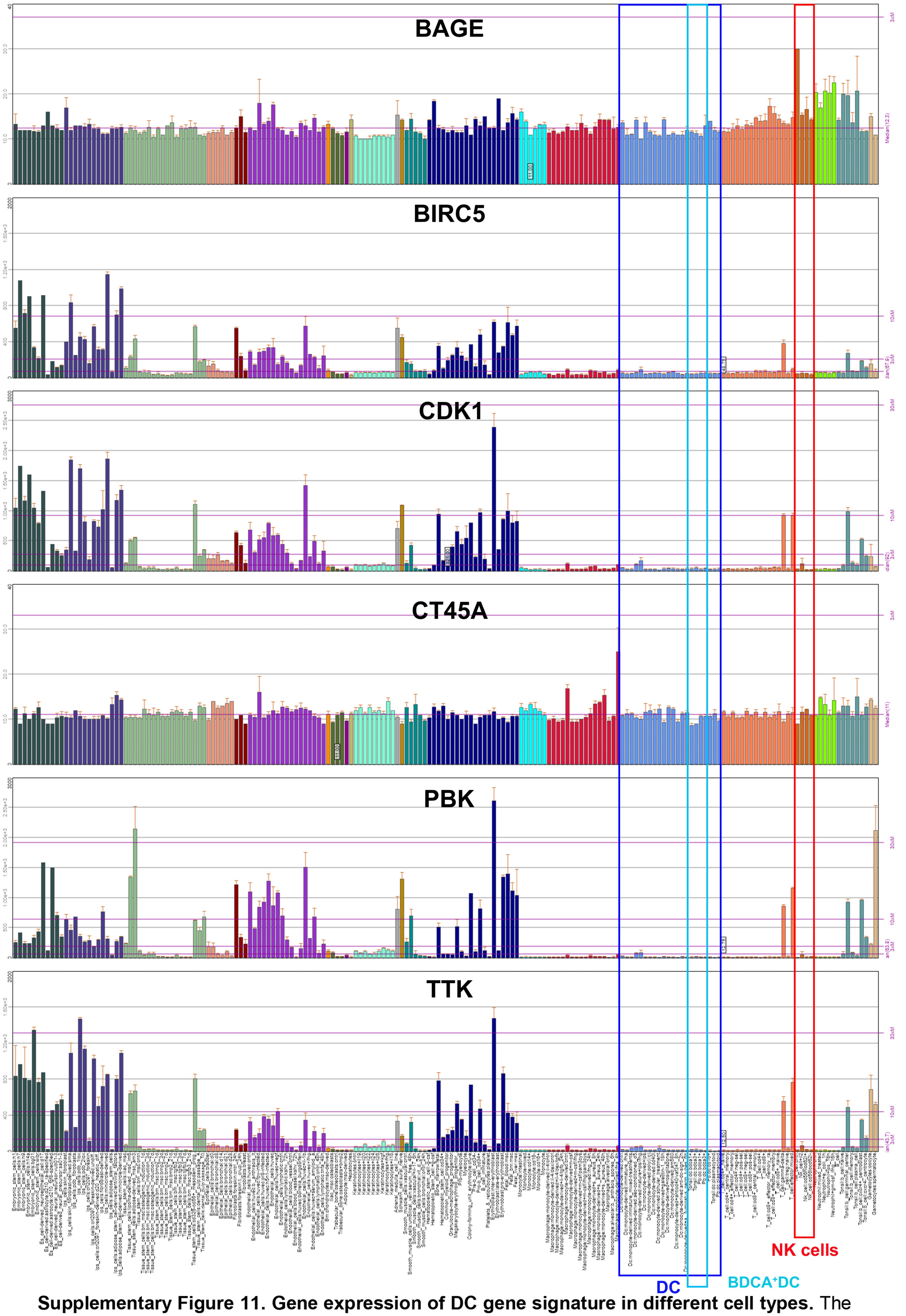

Supplementary Figure 11. Gene expression of DC gene signature in different cell types. The expression of the genes used for the DC gene signature was downloaded from the Primary Cell Atlas database from BioGPS (n=745 independent samples from over 100 separate studies). Expression plotted as GC robust multiarray average values. Total DC populations are in the blue box; BDCA⁺ DC subpopulations are in the light blue box; NK cell populations are in the red box. Data were plotted as bar chart, representing the gene expression average value with error bars as computed by the BioGPS webservice (http://biogps.org/).

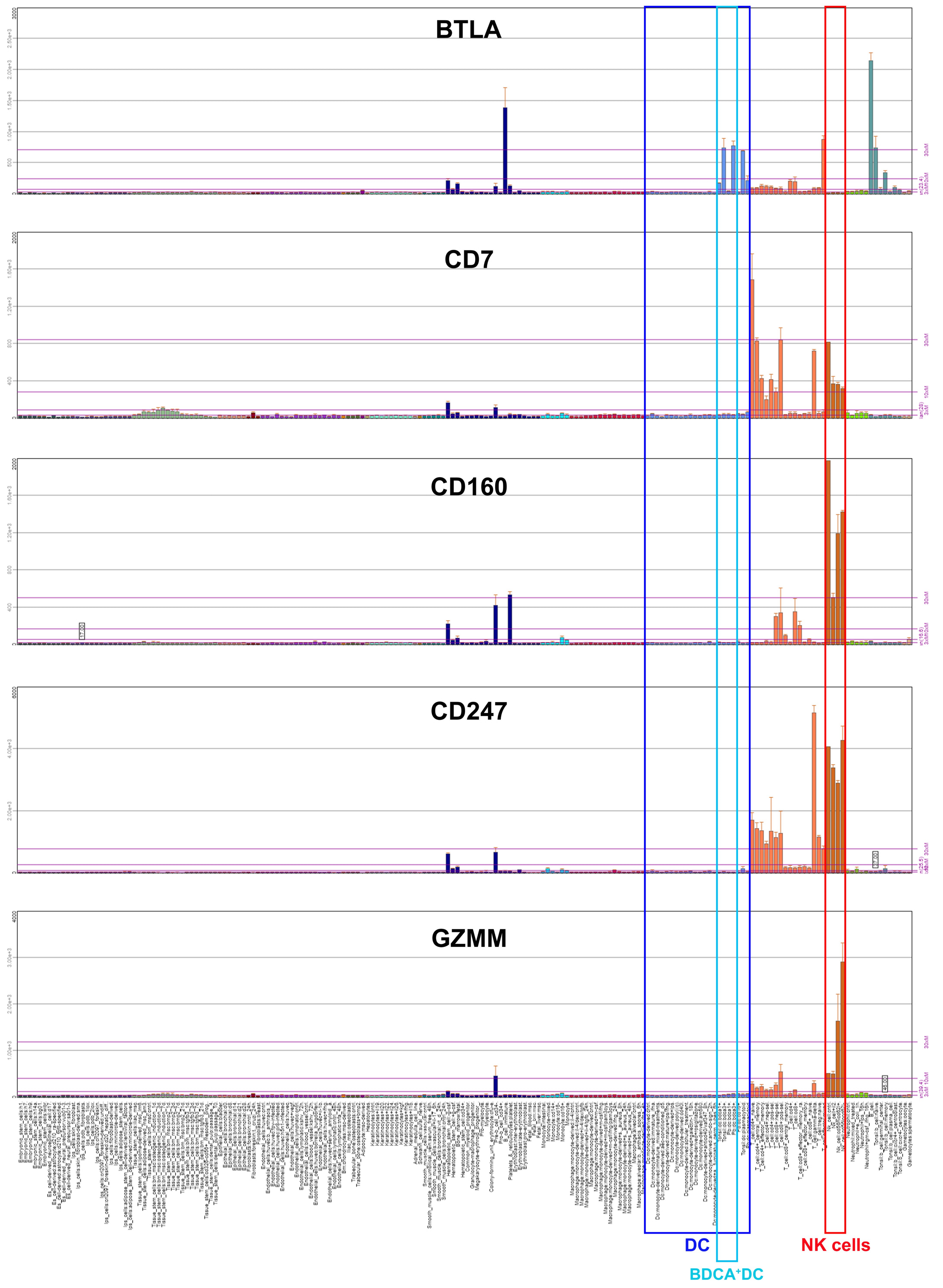

Figure S12 continued

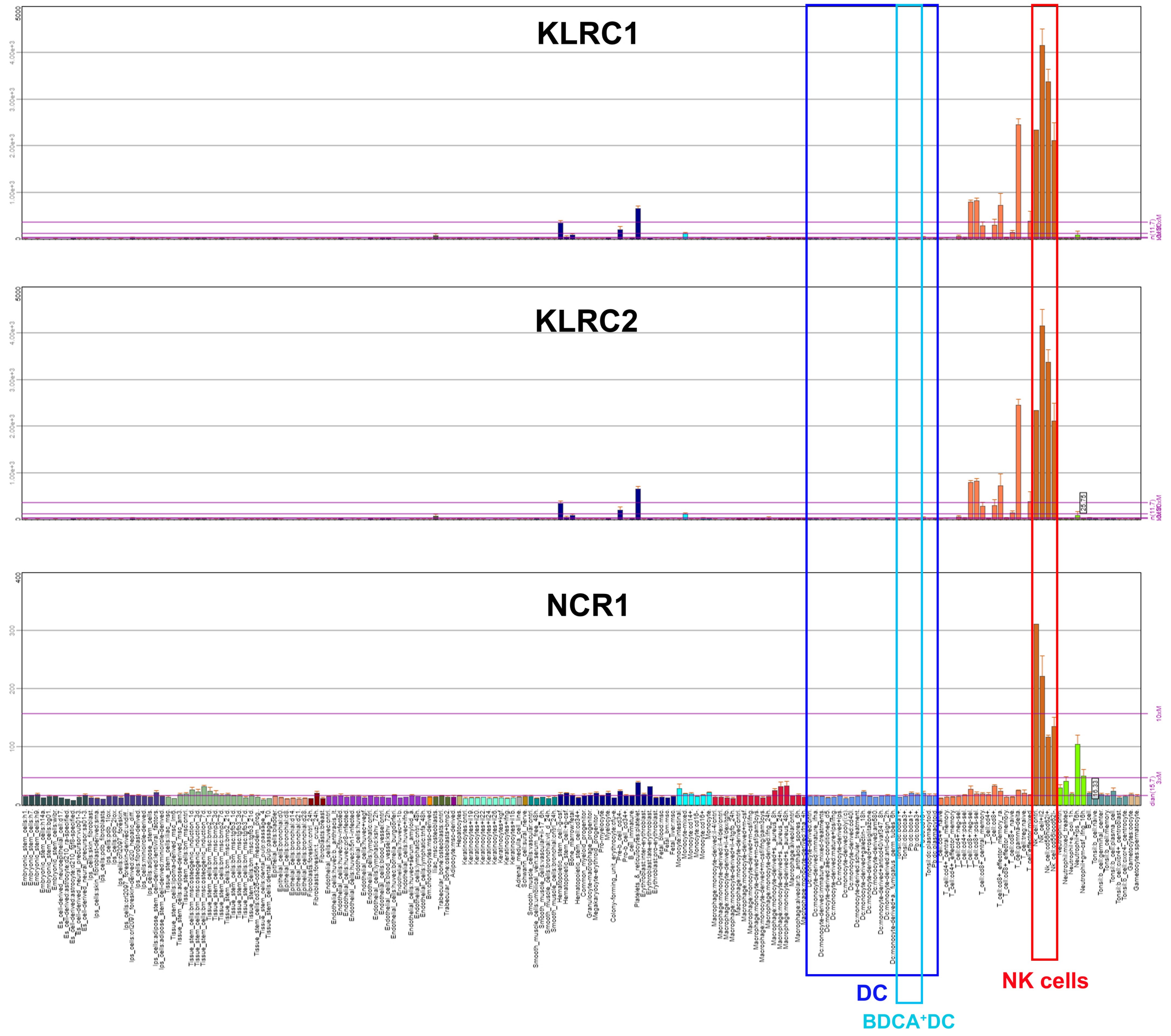

Supplementary Figure 12. Gene expression of NK gene signature in different cell types. The expression of the genes used for the NK gene signature were downloaded from the Primary Cell Atlas database from BioGPS (n=745 independent samples from over 100 separate studies). Expression plotted as GC robust multiarray average values. Total DC populations are in the blue box; BDCA^{+} DC subpopulations are in the light blue box; NK cell populations are in the red box. Data were plotted as bar chart, representing the gene expression average value with error bars as computed by the BioGPS webservice (http://biogps.org/).

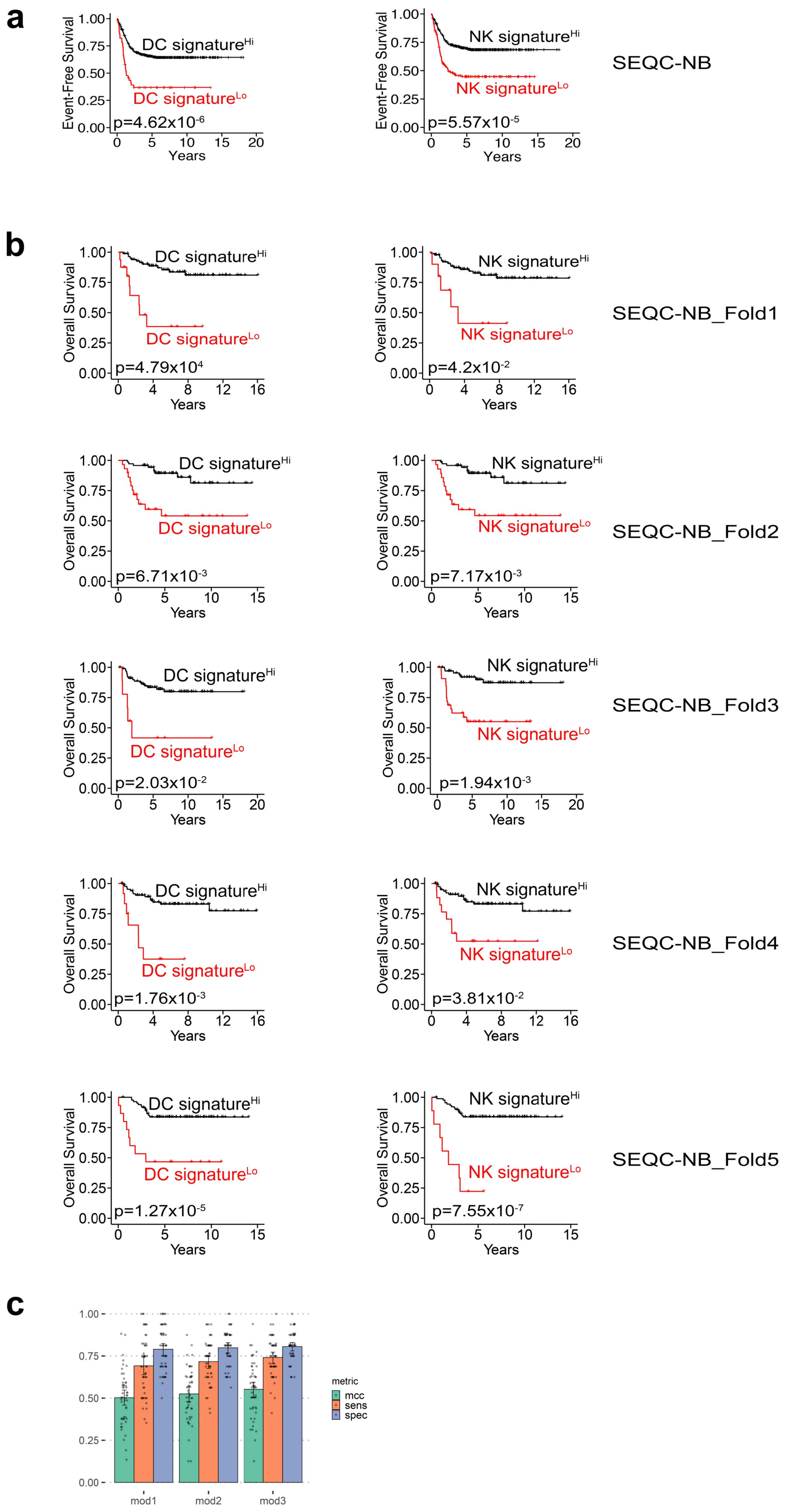

Supplementary Figure 13 related to Figure 6. DC and NK gene signatures positively correlate with increased survival of neuroblastoma patients. a, Kaplan-Meier curves show the duration of event-free survival of SEQC-NB cohort of n=498 patients, according to the DC and NK gene signatures. **b**, Kaplan-Meier curves show the duration of overall survival of SEQC-NB cohort of n=498 patients split in 5 subgroups by a 5-fold cross-validation, according to the DC and NK gene signatures. In a and b, log-rank test with Miller and Siegmund p-value correction was used. **c**, Barplot of average cross-validation Matthews Correlation Coefficient (MCC), sensitivity, and specificity, with 95% confidence intervals, of a logistic regression model predicting OS>2 vs OS<2 yrs, using as predictors: INSS stage, MYCN amplification status, age at diagnosis <18 mo vs >18 mo, CD3E Hi/Lo (mod1); mod1 variables plus the DC signature (mod2); and mod1 variables plus the NK signature (mod3). Each individual barplot refers to n=50 independent data points. The analysis was performed on the SEQC-NB cohort of n=498 patients. Hi = high. Lo = low. Statistically significant p-values are indicated.

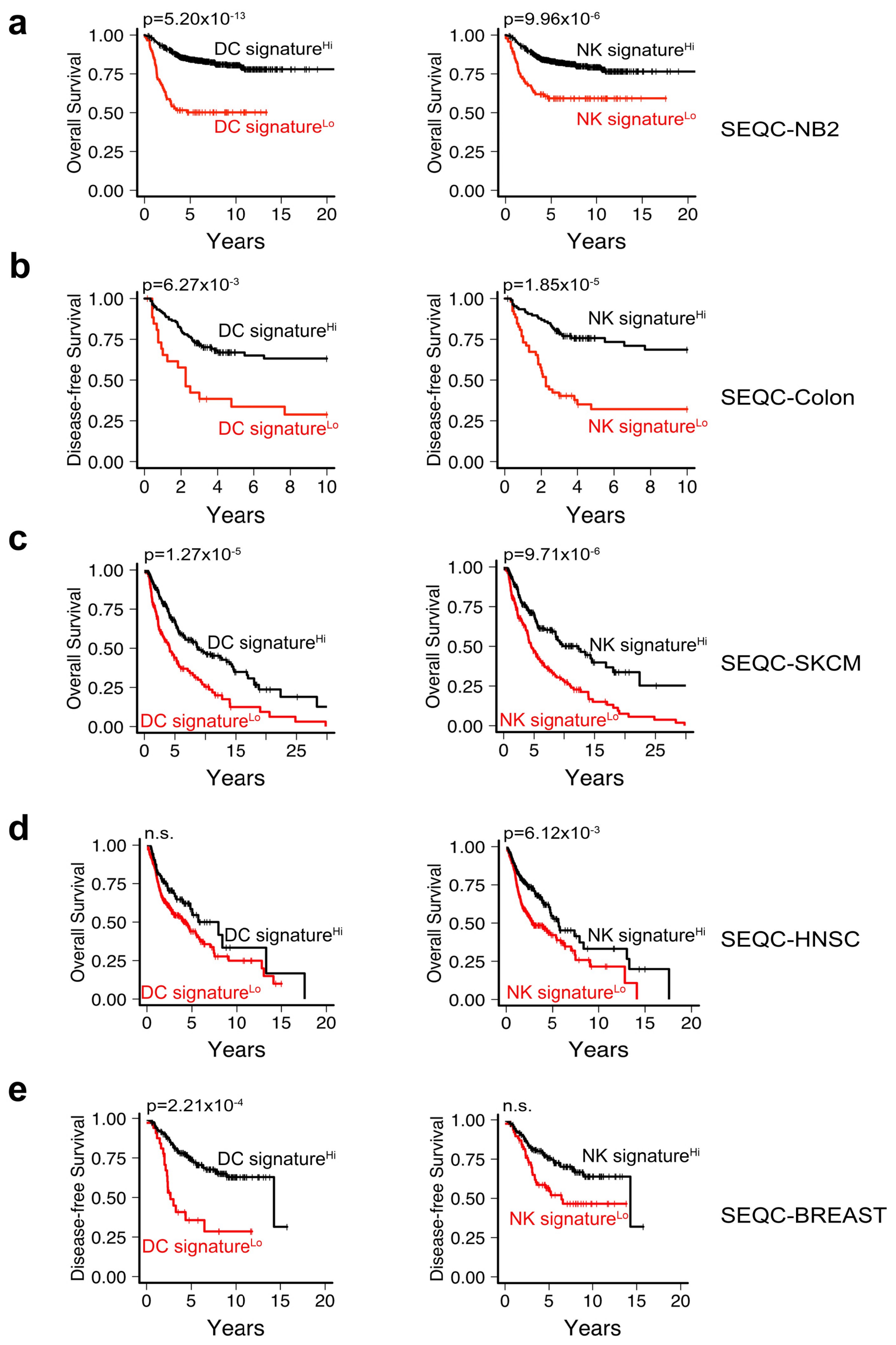

Supplementary Figure 14 related to Figure 6. DC and NK gene signatures positively correlate with increased survival of cancer patients. a-e, Kaplan-Meier curves show the duration of survival of (**a**) NB validation cohort of 649 patients (GEO accession GSE45547), (**b)** colon cancer cohort of 160 patients (GEO accession GSE24551), (**c**) SKCM cohort of 454 patients (https://www.cancer.gov/tcga.), (**d**) HNSC cohort of 520 patients (https://www.cancer.gov/tcga) and (**e**) breast cancer cohort of 266 patients (GEO accession GSE21653) according to the DC and NK gene signatures, respectively. Hi = high. Lo = low. Log-rank test with Miller and Siegmund p-value correction was used. Statistically significant p-values are indicated. n.s. = not significant.

a

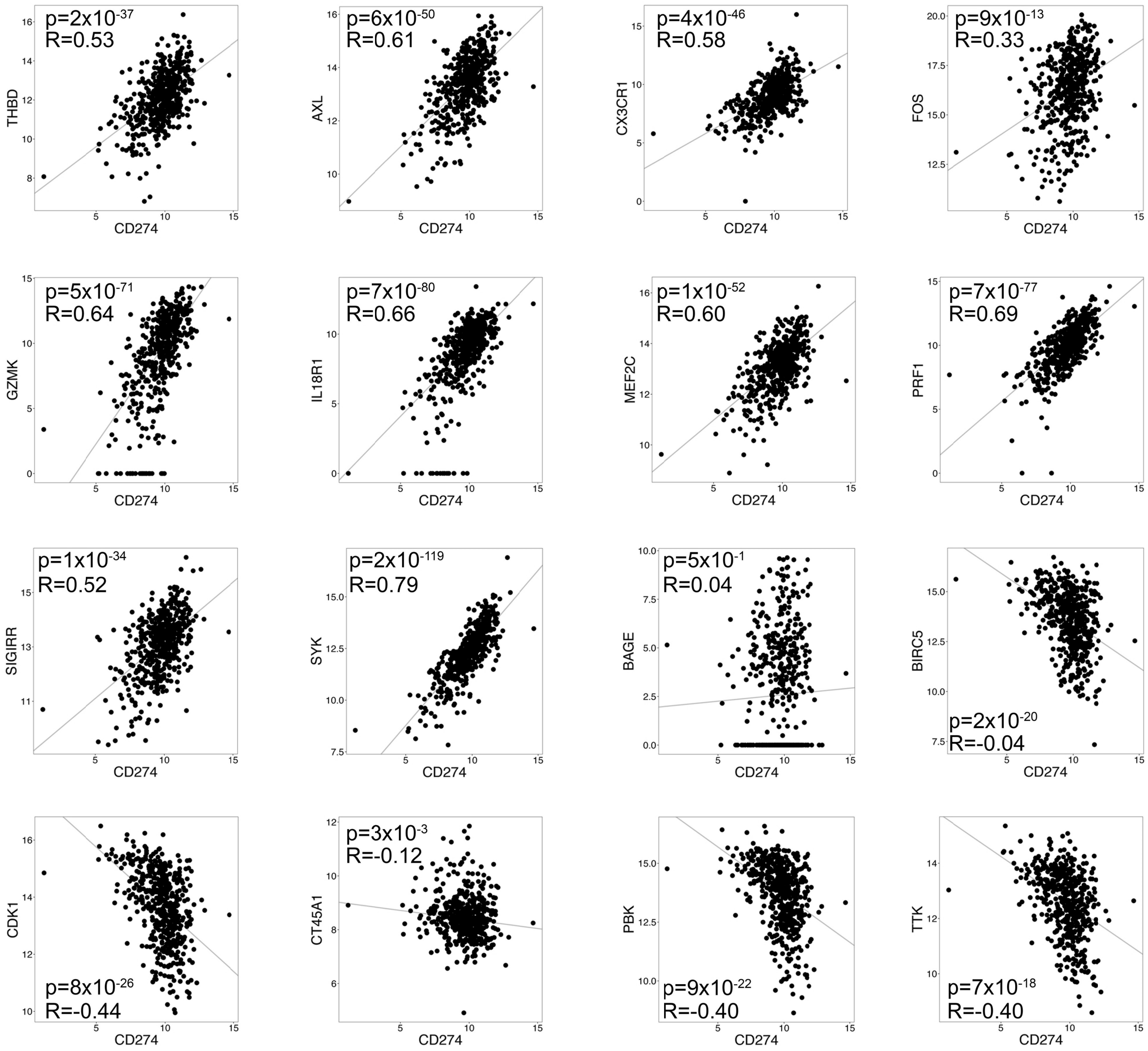

b

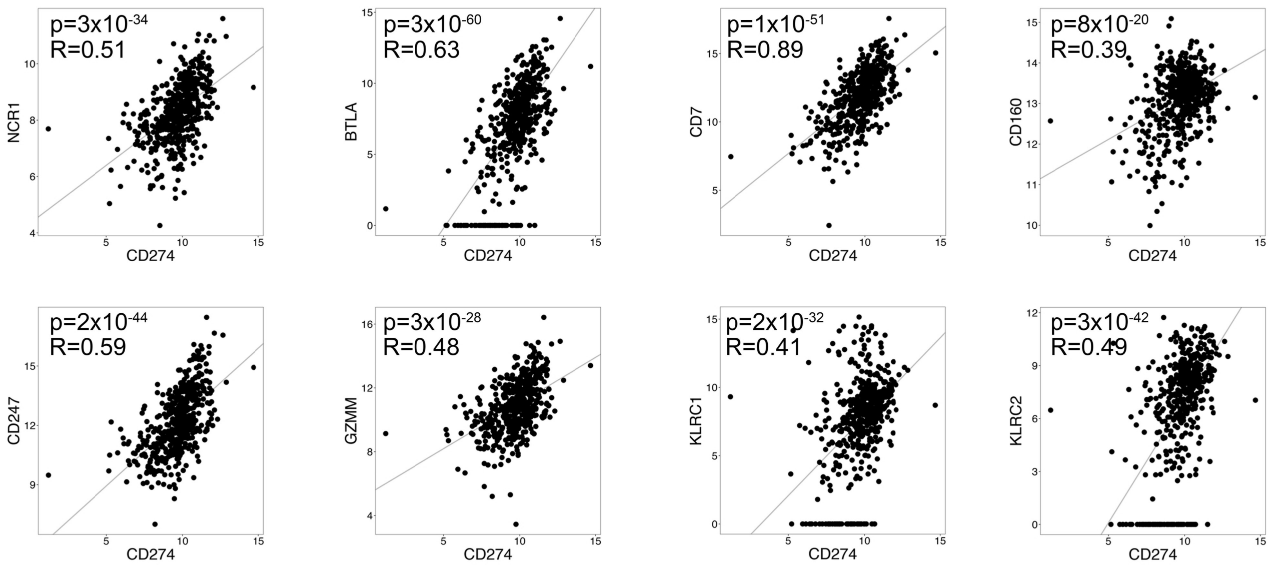

Supplementary Figure 15 related to Figure 6. **a and b**, Correlation of *CD274* (PD-L1) expression with *THBD*- (**a**) and *NCR1* (**b**) associated genes in SEQC-NB (n=498) cohort, analyzed by robust F-test (two-sided). Correlations were assessed measuring the coefficient of determination of a robust linear regression model fit on the data (see Statistical analysis section). No adjustment was required. Statistically significant p-values are indicated.

a

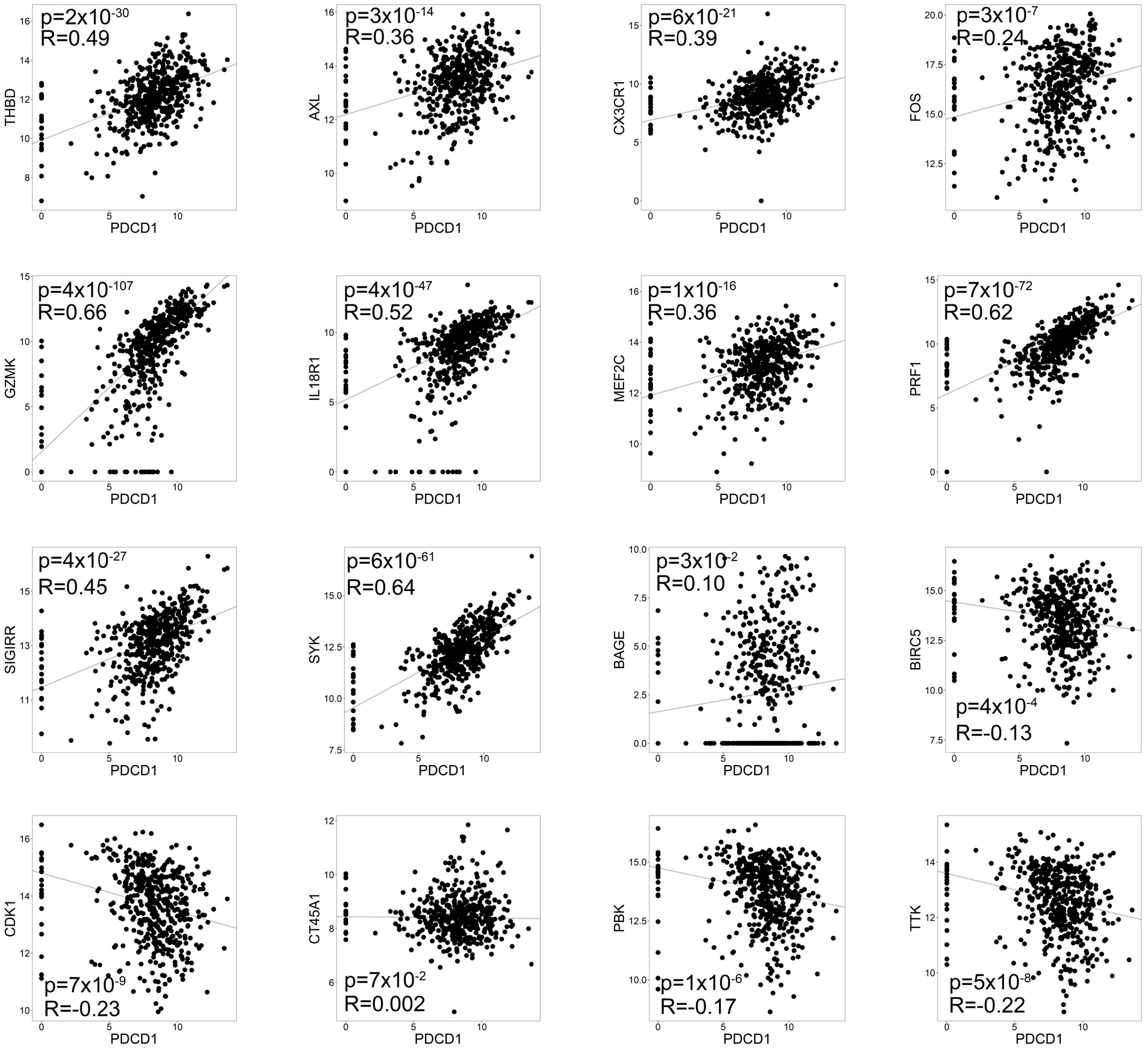

b

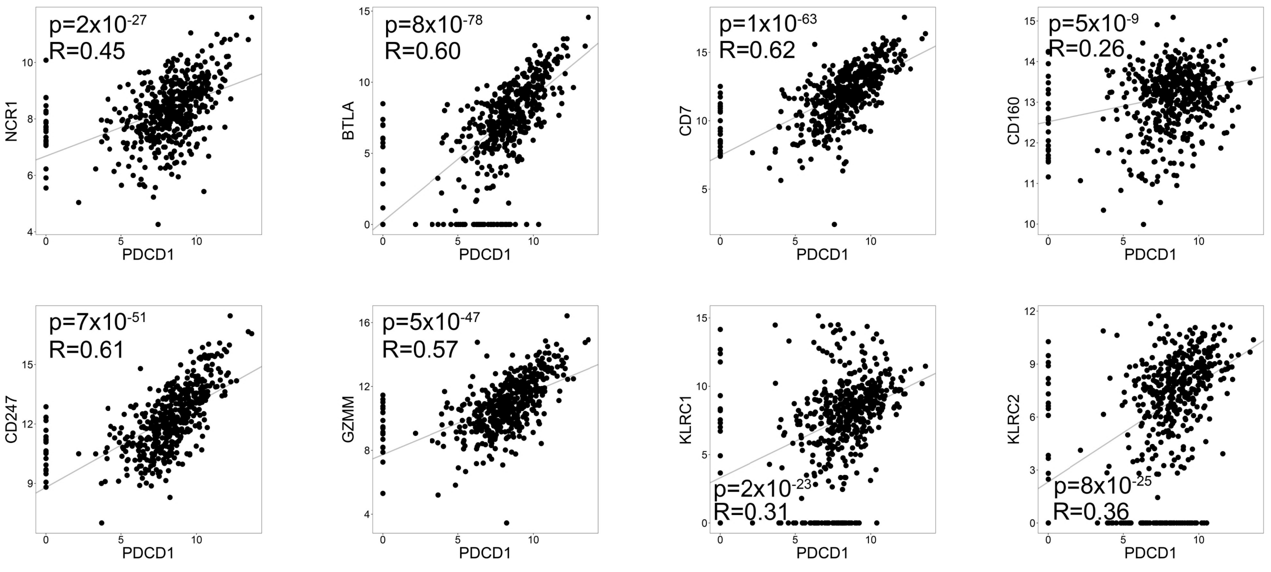

Supplementary Figure 16 related to Figure 6. a and b, Correlation of *PDCD1* (PD-1) expression with *THBD*- (**a**) and *NCR1* (**b**) associated genes in SEQC-NB (n=498) cohort, analyzed by robust F-test (two-sided). Correlations were assessed measuring the coefficient of determination of a robust linear regression model fit on the data (see Statistical analysis section). No adjustment was required. Statistically significant p-values are indicated.

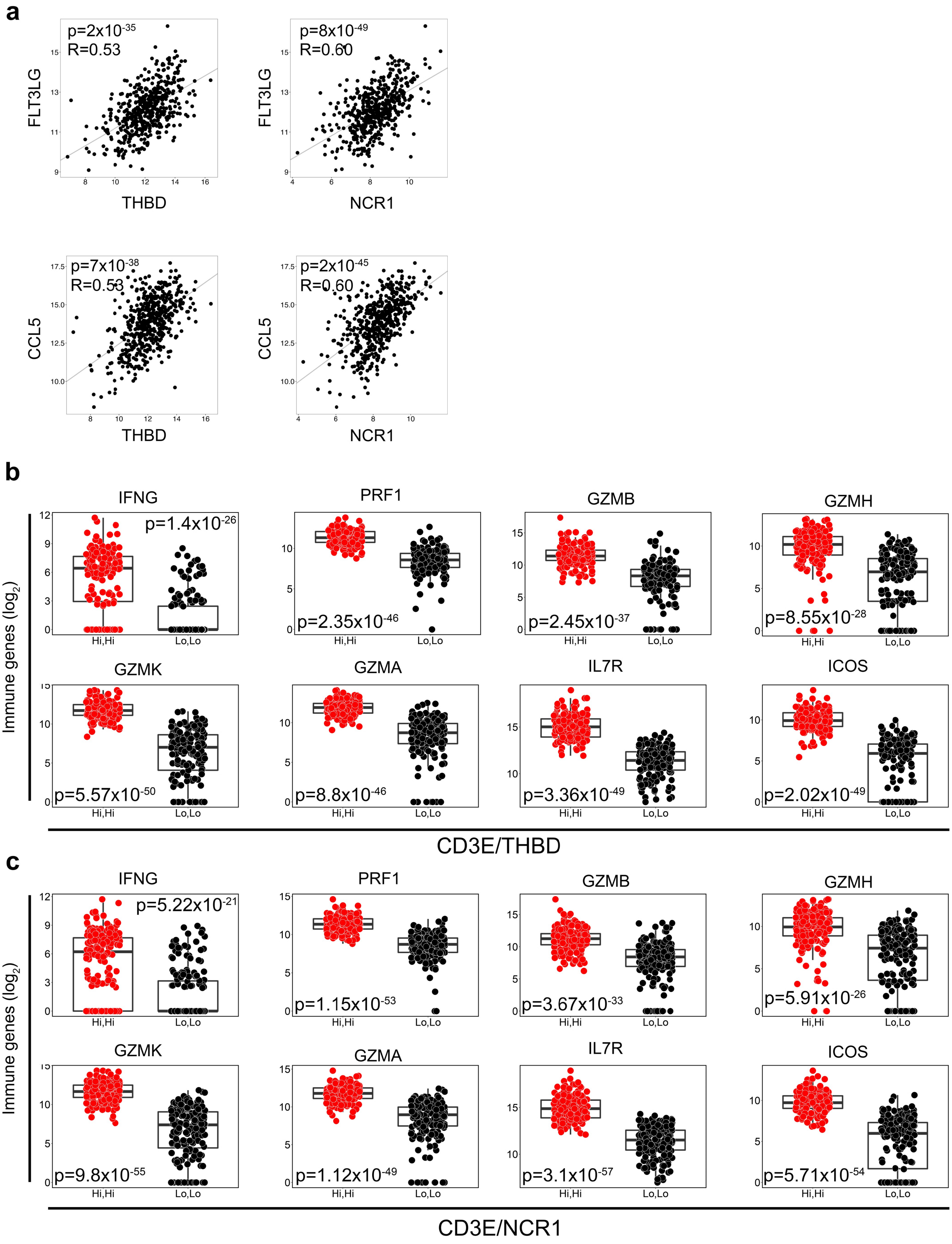

Supplementary Figure 17. a, Correlation of *FLT3LG* and *CCL5* expression with *THBD* and *NCR1* genes in SEQC-NB (n=498) cohort, analyzed by robust F-test (two-sided). Correlations were assessed measuring the coefficient of determination of a robust linear regression model fit on the data (see Statistical analysis section). **b** and **c**, Box plots of gene expression values for the indicated activation and cytotoxicity molecules, according to the high (Hi) and low (Lo) levels of both *CD3E* and *THBD* (**b**) and *CD3E* and *NCR1* (**c**) mRNA (median split), in primary NB lesions from SEQC-NB (n=498) cohorts. Data were analyzed by Kruskal-Wallis rank sum test (two-sided). The boxes show the 25th to 75th percentile; the horizontal lines inside the box represent the median; the upper whisker extends to the largest data point, no more than 1.5 times the IQR from the box; the lower whisker extends to the smallest data point at most 1.5 times the IQR from the box; the dots are individual samples. No adjustment was required. Statistically significant p-values are indicated.

Supplementary Table 1
Clinical and genetic characteristics of 104 newly diagnosed neuroblastoma patients.

Characteristics	Number (%)
Age, months	
Median/range	14/0.3-546
<12	48 (46.2)
≥12, <18	12 (11.5)
≥18	44 (42.3)
Gender	
Male	58 (55.8)
Female	46 (44.2)
INSS stage	
1	29 (27.9)
2	22 (21.15)
3	7 (6.7)
4	37 (35.6)
4S	9 (8.65)
INRG stage	
L1	46 (44.3)
L2	12 (11.5)
M	37 (35.6)
MS	9 (8.6)
Histologic classification (INPC or Shimada)	
Favorable	72 (70)
Unfavorable	32 (30)
Grade of NB differentiation (INPC or Shimada)	
Differentiating	21 (20.2)
Undifferentiating	83 (79.8)
MYCN status	
Not amplified	68 (65.5)
Gain	12 (11.5)
Amplified	24 (23)
1p	
Normal	83 (79.8)
Deletion	21 (20.2)
