# Supplementary material for: Cellular and gene signatures of tumor-infiltrating dendritic cells and natural-killer cells predict prognosis of neuroblastoma
Source: Nat Commun. 2020 Nov 25;11:5992. doi: 10.1038/s41467-020-19781-y (PMC7689423; doi:10.1038/s41467-020-19781-y)
Supplement: Supplementary file 3 — Description of Additional Supplementary Files [file 41467_2020_19781_MOESM3_ESM.pdf]

Description of Additional Supplementary files

Supplementary Data 1: Differently expressed immune genes in high-CD3E NBs from both SEQC-NB (n=498) and Nanostring-NB (n=36) cohorts.

Supplementary Data 2: Gene categories from Reactome analysis performed on both SEQC-NB (n=498) and Nanostring-NB (n=36) cohorts.

Supplementary Data 3: Immune gene signature analysis on both SEQC-NB (n=498) and Nanostring-NB (n=36) cohorts.

Supplementary Data 4: Fold change, p-value, q-value and hazard ration of THBD- and NCR1-related genes.

Supplementary Data 5: 10 iterations of 5-fold Cross Validation (10 x 5 fold) on DC and NK gene signatures.

Supplementary Data 6: MCC, sensitivity and specificity related to DC and NK gene signatures.
