# Supplementary material for: Cellular and gene signatures of tumor-infiltrating dendritic cells and natural-killer cells predict prognosis of neuroblastoma
Source: Nat Commun. 2020 Nov 25;11:5992. doi: 10.1038/s41467-020-19781-y (PMC7689423; doi:10.1038/s41467-020-19781-y)
Supplement: Supplementary file 10 — Reporting Summary [file 41467_2020_19781_MOESM10_ESM.pdf]

nature research

Corresponding author(s): Doriana Fruci

Last updated by author(s): Sep 25, 2020

Reporting Summary

Nature Research wishes to improve the reproducibility of the work that we publish. This form provides structure for consistency and transparency in reporting. For further information on Nature Research policies, see our Editorial Policies and the Editorial Policy Checklist.

Statistics

For all statistical analyses, confirm that the following items are present in the figure legend, table legend, main text, or Methods section.

n/a	Confirmed	
☐	☒	The exact sample size (*n*) for each experimental group/condition, given as a discrete number and unit of measurement
☐	☒	A statement on whether measurements were taken from distinct samples or whether the same sample was measured repeatedly
☐	☒	The statistical test(s) used AND whether they are one- or two-sided<br>*Only common tests should be described solely by name; describe more complex techniques in the Methods section.*
☐	☒	A description of all covariates tested
☐	☒	A description of any assumptions or corrections, such as tests of normality and adjustment for multiple comparisons
☐	☒	A full description of the statistical parameters including central tendency (e.g. means) or other basic estimates (e.g. regression coefficient) AND variation (e.g. standard deviation) or associated estimates of uncertainty (e.g. confidence intervals)
☐	☒	For null hypothesis testing, the test statistic (e.g. *F*, *t*, *r*) with confidence intervals, effect sizes, degrees of freedom and *P* value noted<br>*Give P values as exact values whenever suitable.*
☒	☐	For Bayesian analysis, information on the choice of priors and Markov chain Monte Carlo settings
☐	☒	For hierarchical and complex designs, identification of the appropriate level for tests and full reporting of outcomes
☐	☒	Estimates of effect sizes (e.g. Cohen's *d*, Pearson's *r*), indicating how they were calculated

Our web collection on statistics for biologists contains articles on many of the points above.

Software and code

Policy information about availability of computer code

Data collection	No software was used for the data collection of this study.
Data analysis	A description of the software and code has been included in Methods. Preprocessing of raw data as well as all subsequent statistical and survival analyses were performed in the R environment for statistical computing (version 3.6.2), using libraries available on CRAN or Bioconductor repositories, as detailed in Methods. Scanned images were viewed and captured with Hamamatsu Photonics's image viewer software (NDP.view2 Viewing software U12388-01). Confocal imaging was captured with an Olympus Fluoview FV1000 confocal microscope equipped with FV10-ASW 4.1 software.

For manuscripts utilizing custom algorithms or software that are central to the research but not yet described in published literature, software must be made available to editors and reviewers. We strongly encourage code deposition in a community repository (e.g. GitHub). See the Nature Research guidelines for submitting code & software for further information.

Data

Policy information about availability of data

All manuscripts must include a data availability statement. This statement should provide the following information, where applicable:

- Accession codes, unique identifiers, or web links for publicly available datasets
- A list of figures that have associated raw data
- A description of any restrictions on data availability

The authors declare that all data supporting the findings of this study are available within the paper and its supplementary information files. Bioinformatics investigation was performed by querying gene expression data from publicly available gene expression cancer datasets from GEO GSE62564 and GSE45547 for neuroblastoma (DOIs: 10.1186/s13059-015-0694-1 and 10.1038/cddis.2013.84, respectively), GSE24551 for colorectal cancers (DOI: 10.1136/gutjnl-2011-301179), GSE21653 for breast cancers (DOI: 10.1007/s10549-010-0897-9), and from TCGA Research Network (https://www.cancer.gov/tcga) for SKCM and HNSC. The gene

interaction network is based on gene-gene interaction information of the STRING database and was generated via the query interface on the webpage (http://string-db.org). The Primary Cell Atlas database from BioGPS (http://biogps.org) was used to refine the DC and NK cell gene signatures. Any other relevant data and code is available from the corresponding authors upon reasonable request.

Field-specific reporting

Please select the one below that is the best fit for your research. If you are not sure, read the appropriate sections before making your selection.

☒ Life sciences ☐ Behavioural & social sciences ☐ Ecological, evolutionary & environmental sciences

For a reference copy of the document with all sections, see nature.com/documents/nr-reporting-summary-flat.pdf

Life sciences study design

All studies must disclose on these points even when the disclosure is negative.

Sample size	Our NB cohort is of n=104. This cohort was collected during a long time and we believe it is sufficient to ask the questions we put forward in the manuscript. In addition, 36 in-house NB samples were profiled with Nanostring and cohorts where data are available in public repositories were also used, i.e. GSE62564: n=498; GSE45547: n=649; GSE24551: n=160; GSE21653: 266; TCGA SKCM: n= 454; TCGA HNSC n= 520. Total = 2651 samples.
Data exclusions	No data were excluded.
Replication	No replication was done, but validation cohorts were analysed. All data obtained from the nanostring cohort were validated in the public GEO neuroblastoma GSE62564 dataset (DOIs: 10.1186/s13059-015-0694-1. The derived DC and NK gene signatures were firmly validated in several different datasets across multiple gene expression platforms described above.
Randomization	For each marker (genes: CD3E, CD8A, CD4, THBD and NCR1; proteins: CD141 and NKp46), the median of their distribution was used as cut-off to stratify all patients. Patients were also stratified according to the INRG stage, MYCN amplification status and age at diagnosis. The Kaplan–Meier method was used for the estimation of overall survival and event-free survival curves. The log-rank test, as implemented in the survival R package, was used to compare OS and EFS between different groups of patients. Survival analysis was performed in a univariate fashion, feature by feature. In the case of continuous variables, unless otherwise specified, the optimal threshold yielding the best dichotomic patient stratification was selected. In experiments involving machine learning models, patients were split into train and test sets using a k-fold cross-validation schema.
Blinding	Immunostaining evaluation, i.e., immunohistochemistry and immunofluorescence analyses, were done blinded by two independent examiners of which one was a board-certified pathologist. All data analyses were performed blindly, independently of sample knowledge.

Reporting for specific materials, systems and methods

We require information from authors about some types of materials, experimental systems and methods used in many studies. Here, indicate whether each material, system or method listed is relevant to your study. If you are not sure if a list item applies to your research, read the appropriate section before selecting a response.

Materials & experimental systems

n/a	Involved in the study	
☐	☒	Antibodies
☒	☐	Eukaryotic cell lines
☒	☐	Palaeontology and archaeology
☒	☐	Animals and other organisms
☐	☒	Human research participants
☒	☐	Clinical data
☒	☐	Dual use research of concern

Methods

n/a	Involved in the study	
☒	☐	ChIP-seq
☒	☐	Flow cytometry
☒	☐	MRI-based neuroimaging

Antibodies

Antibodies used	The following antibodies were used: anti-mouse monoclonal CD141 (Leica Biosystems, cat.n. NCL-CD141, clone 15C8, lot 6034959), mouse monoclonal Human NKp46/NCR1 (R&D Systems, cat. n. MAB1850-100, clone 195314, lot JRA0216071), mouse monoclonal Alexa Fluor® 647 anti-human CD8a (BioLegend, cat. n. 372906, clone C8/144B, lot B239675), mouse monoclonal Thrombomodulin (Invitrogen, cat. n. MA5-11454, clone 141C01-1009, lot UB2723074), rabbit polyclonal CD335 (NKp46) (Invitrogen, cat. n. PA5-87271, lot UE2779132A), Alexa Fluor 555 F(ab')2-Goat anti-Rabbit IgG (H+L) (Invitrogen cat. n. A21430, lot 2016414), Alexa Fluor 488 F(ab')2-Goat anti-Mouse IgG (H+L) (Invitrogen cat. n. A11017, lot 2108802).
Validation	Antibodies used in this study are well validated. This is clearly demonstrated by the manufacturer as well as the vast number of citations refereing to the antibodies. The specificity of each antibody was also validated on FFPE tonsil sections as positive control.

For anti-mouse monoclonal CD141 (clone 15C8) certified manufacturing facilities from the company guarantee full quality control. This reagent has been prepared from the supernatant of cell culture. Simian COS cells were transfected with the expression vector pSV2 containing thrombomodulin cDNA synthesized immunoreactive and functionally active thrombomodulin. Its use is recommended for immunohistochemistry on paraffin setions.
For mouse monoclonal human NKp46/NCR1 (clone 195314) certified manufacturing facilities by the company guarantee full quality control. This reagent has been prepared from mouse T cell hybridoma transfected with human NKp46/NCR1. It is recommended for several applications, i.e., Western Blot, Flow Cytometry, Immunohistochemistry, Agonist Activity and CyTOF as reported in Horowitz, A. et al. Sci. Transl. Med. (2013) 208ra145. NKp46/NCR1 was detected in immersion fixed NK-92 human natural killer lymphoma cell line and human peripheral blood mononuclear cells using 25 µg/mL of this antibody for 3 hours at room temperature. NKp46/NCR1 was detected in immersion fixed paraffin-embedded sections of human tonsil using 5 µg/mL of this antibody for 1 hour at room temperature.
For mouse monoclonal Alexa Fluor® 647 anti-human CD8a (clone C8/144B) ,certified manufacturing facilities from the company guarantee full quality control. A 13 amino acid synthetic peptide from the C-terminal cytoplasmic domain of the alpha chain of the human CD8 molecule constitutes the immunogen. Each lot of this antibody is quality control tested for flow cytometric staining and immunofluorescence staining on paraffin embebbed tissues.
For mouse monoclonal Thrombomodulin (clone 141C01-1009) certified manufacturing facilities from the company guarantee full quality control. This antibody targets CD141 in Immunohistochemistry, Immunocytochemistry, Immunofluorescence and Western Blot applications and shows reactivity with Human and Rat samples. Its immunogen is recombinant protein encoding the six repeated EGF domains of human thrombomodulin.
For rabbit polyclonal CD335 (NKp46) certified manufacturing facilities from the company guarantee full quality control. Its use is recommended for Immunocytochemistry, Immunofluorescence and Western Blot. Its immunogen is the recombinant full length Human NKp46. Immunofluorescence analysis of NKp46 has been performed in U2OS cells followed by DAPI for nuclear staining, with evident membrane staining.

Human research participants

Policy information about studies involving human research participants

Population characteristics	Tumor samples from 104 neuroblastoma patients collected at diagnosis and prior to any therapy were used. They were 58 male and 46 female. Age at diagnosis was <12 months for 48 patients, between 12 and 18 months for 12 patients and ≥18 months for 44 patients. For each patient, written informed parental consent was obtained in accordance with the Declaration of Helsinki. All available characteristics are reported in Supplementary Table 4.
Recruitment	In-house neuroblastoma cohort was collected prospectively from 2002-2017 at the Dept of Oncohaematology, Bambino Gesù Children's Hospital in Italy. Only patients where sufficient tissue was available were included in the study.
Ethics oversight	The study was approved by the Ethical Committee of the Bambino Gesù Children's Hospital. Cod. n. 1337_OPBG.

Note that full information on the approval of the study protocol must also be provided in the manuscript.
